# Analyzing gait data measured by wearable cyborg hybrid assistive limb during assisted walking: gait pattern clustering

**DOI:** 10.3389/fmedt.2024.1448317

**Published:** 2024-12-16

**Authors:** Yasuko Namikawa, Hiroaki Kawamoto, Akira Uehara, Yoshiyuki Sankai

**Affiliations:** ^1^ Degree Programs in Systems and Information Engineering, University of Tsukuba, Tsukuba, Japan; ^2^ Institute of Systems and Information Engineering, University of Tsukuba, Tsukuba, Japan; ^3^ Center for Cybernics Research, University of Tsukuba, Tsukuba, Japan; ^4^ CYBERDYNE, Inc., Tsukuba, Japan

**Keywords:** hybrid assistive limb (HAL), cybernics treatment, wearable devices, gait analysis, hierarchical clustering, neuromuscular diseases

## Abstract

**Introduction:**

The wearable cyborg Hybrid Assistive Limb (HAL) is a therapeutic exoskeletal device that provides voluntary gait assistance using kinematic/kinetic gait data and bioelectrical signals. By utilizing the gait data automatically measured by HAL, we are developing a system to analyze the wearer's gait during the intervention, unlike conventional evaluations that compare pre- and post-treatment gait test results. Despite the potential use of the gait data from the HAL's sensor information, there is still a lack of analysis using such gait data and knowledge of gait patterns during HAL use. This study aimed to cluster gait patterns into subgroups based on the gait data that the HAL automatically collected during treatment and to investigate their characteristics.

**Methods:**

Gait data acquired by HAL, including ground reaction forces, joint angles, trunk angles, and HAL joint torques, were analyzed in individuals with progressive neuromuscular diseases. For each measured item, principal component analysis was applied to the gait time-series data to extract the features of the gait patterns, followed by hierarchical cluster analysis to generate subgroups based on the principal component scores. Bayesian regression analysis was conducted to identify the influence of the wearer's attributes on the clustered gait patterns.

**Results:**

The gait patterns of 13,710 gait cycles from 457 treatments among 48 individuals were divided into 5–10 clusters for each measured item. The clusters revealed a variety of gait patterns when wearing the HAL and identified the characteristics of multiple sub-group types. Bayesian regression models explained the influence of the wearer's disease type and gait ability on the distribution of gait patterns to subgroups.

**Discussion:**

These results revealed key differences in gait patterns related to the wearer's condition, demonstrating the importance of monitoring HAL-assisted walking to provide appropriate interventions. Furthermore, our approach highlights the usefulness of the gait data that HAL automatically measures during the intervention. We anticipate that the HAL, designed as a therapeutic device, will expand its role as a data measurement device for analysis and evaluation that provides gait data simultaneously with interventions, creating a novel cybernics treatment system that facilitates a multi-faceted understanding of the wearer's gait.

## 1 Introduction

Gait is achieved by control via neural signals from the central nervous system to effector muscles based on motor intention (1, 2). Degeneration of the neuromuscular system, as seen in neuromuscular diseases, affects these signal pathways responsible for movement, leading to gait disorders (2, 3). For a long time, many rehabilitation programs for such gait disorders focused on compensatory strategies using residual function, rather than directly aiming to restore function (2). However, in recent years, a new treatment approach, that target the neuromuscular system for functional improvement and regeneration have been attracting attention.

The world's first wearable cyborg Hybrid Assistive Limb (HAL; CYBERDYNE Inc., Tsukuba, Japan) is a therapeutic device that assists the wearer's gait through a voluntary drive (4, 5). To achieve movement under fusion with the wearer, the HAL generates assistive torques using sensor information, such as bioelectrical signals, which reflect the wearer's motor intentions, and ground reaction forces, which represent the gait phase (6, 7). Walking with HAL creates a neural information transfer loop between the central nervous system and the musculoskeletal system via HAL, which is believed to be important for neuroregeneration (2, 5). Cybernics treatment, an intervention using HAL, strengthens the neural loop via the device through repetition without increasing the neuromuscular system's excessive load and fatigue of the wearer. Specifically, HAL assists the wearer's movements based on their motor intentions, facilitating the synchronization of motor commands and sensory feedback at appropriate timings, which forms interactive biofeedback loops. The repetition of this process, which enhances the transmission efficiency of synapses, is a crucial aspect of cybernics treatment (2, 8–10). Previous studies have demonstrated that this approach is effective in maintaining and improving gait ability in individuals with gait disorders caused by progressive neuromuscular diseases (2, 11–14).

To monitor the effect and progress of intervention using HAL, conventional assessments in cybernics treatment compare some indicators of gait ability, such as the 2-min or 6-min walk distance, 10-meter walk speed, and Functional Ambulation Category (FAC), before and after a series of several intervention sessions (2, 11–17). These metrics are measured without HAL and are performed separately from treatment. Although these conventional methods are effective in examining intervention-related changes in gait ability, they are difficult to use in daily practice because of the high burden of measurement (18). The physical strain on the patients to perform gait tests for evaluation only, separate from treatment, the burden on the clinical operators to measure data while ensuring patient safety, and time constraints make it practically difficult to perform these measurements at a high frequency. Moreover, current methods are disconnected from the process of the actual treatment sessions, which does not bring objective information on the wearer's gait condition during intervention. Since the core of HAL-assisted gait is the feasibility of repeatedly forming interactive biofeedback loops even in individuals who struggle with this process on their own, it is desirable to monitor the gait resulting from the interaction between the HAL and the wearer; however, there is no established method for this.

**Figure 1:**
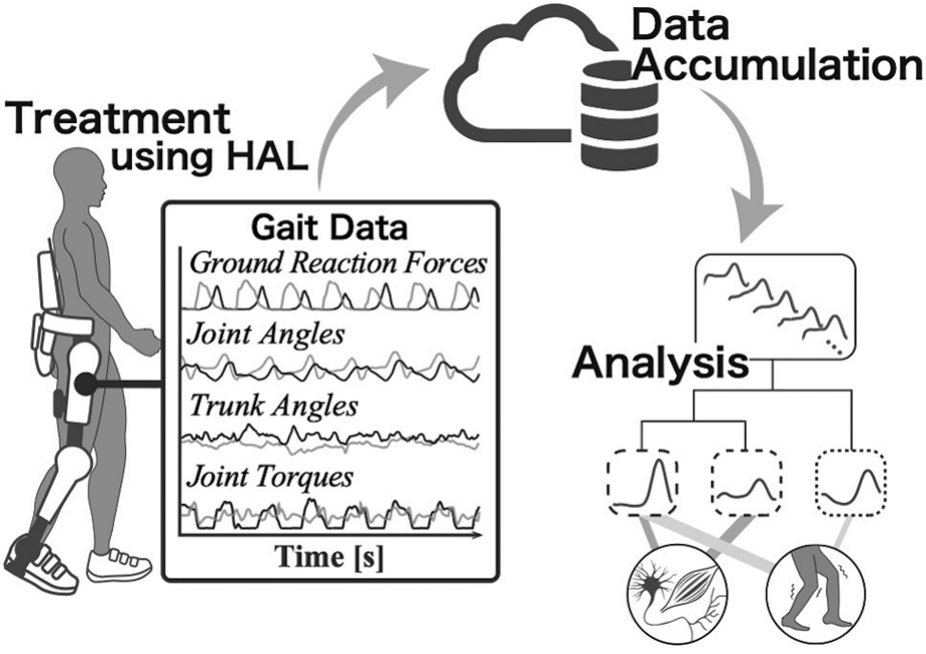
Conceptual diagram of the use of gait data measured by HAL. The process includes treatment, data accumulation, and analysis using HAL. In addition to the conventional intervention using HAL, a more sophisticated treatment system will be constructed by using the gait time-series data that HAL automatically measures during the assistance.

Therefore, we have begun developing a system for data accumulation and analysis in cybernics treatment that utilizes the gait data measured by the HAL while it assists the wearer's gait (Figure 1). In addition to the bioelectrical signals and ground reaction forces required for torque generation, HAL acquires gait data on lower limb joint angles, trunk angles, and joint torques of the HAL during the intervention. The use of the HAL sensor to collect data for analysis and evaluation has significant advantages in clinical applications. These gait time-series data are automatically recorded, without creating an additional burden for patients and clinical operators beyond the treatment itself, nor altering the procedural flow of existing clinical practices. The HAL use also facilitates the accumulation and analysis of data from various cases more efficiently than conventional methods. This creates a foundation for the efficient aggregation of consistent and objective data obtained through a common device, HAL, which is particularly beneficial in cybernics treatment, as it is sometimes applied to rare diseases. Additionally, it enables the implementation and evaluation of a treatment that considers gait motion during the intervention in each session, addressing questions about the wearer's gait condition during the intervention and allowing for more frequent evaluations in every treatment session compared to conventional methods. Moreover, this system not only offers benefits in clinical settings but also introduces a new role for HAL as a device for data measurement, beyond just being a therapeutic device. It will serve as a key framework in cybernics treatment for capturing the wearer's gait condition associated with HAL intervention from multiple perspectives.

Despite the potential utilization of gait data during the intervention, research on the gait while wearing HAL remains limited (18–20). Puentes et al. (19) evaluated gait coordination with planar covariation in hemiparetic stroke patients by recording segmental kinematics while wearing HAL using motion capture, although it only covered the coordination between the paretic and non-paretic legs. Kadone et al. (20) analyzed muscular activation patterns and synergies during HAL intervention in an individual with myelopathy, but the data was recorded using EMG sensors separate from the HAL sensors and it was a single case report focusing only on muscular activities. Our former study (18) assessed bioelectrical signal patterns measured by HAL, primarily comparing the similarity of patterns between those without gait disorder and those with neuromuscular diseases, without addressing kinematic and kinetic data other than bioelectrical signals or examining pattern characteristics and differences among individuals undergoing treatment. The lack of focused studies on the kinematics and kinetics of gait during the intervention, including studies using gait data automatically acquired by HAL, hinders our understanding of how patients walk under fusion with HAL in daily clinical settings. Addressing this gap is crucial for evaluating gait with metrics that capture the characteristics of HAL-assisted walking, and for creating optimal feedback and treatment strategies for each individual.

The assistive torques provided by the HAL through voluntary control vary according to the gait motions generated by the wearer. Consequently, the gait patterns produced by the interaction between the HAL and the wearer differ according to the gait cycle, treatment session, individual, and the wearer's gait ability. Subgrouping such diverse gait patterns through clustering contributes to identifying typical gait types and systematically characterized gait features, which lead to effective evaluations and intervention strategies (21–23). Similarly in cybernics treatment, clustering various gait patterns and knowing their relationship to the wearer's disease type, gait ability, and other factors provides new information for identifying the specific characteristics of the wearer's gait and developing treatment plans. Our unique approach of utilizing the automatically measured gait data from HAL has facilitated the accumulation of large volumes of gait data, enabling this study to conduct a pioneering comprehensive clustering analysis of kinematic and kinetic patterns during interventions for rare neuromuscular diseases.

The objective of this study was to cluster the gait patterns of individuals with neuromuscular diseases receiving cybernics treatment into subgroups based on gait kinematic and kinetic data automatically measured by the HAL during assisted walking, followed by examining the features of these subgroups, as part of a novel approach to establish a method of multi-faceted gait understanding while cybernics treatment. The clustering results characterized the patterns of ground reaction forces, lower limb joint angles, trunk angles, and joint torque of the HAL during walking with HAL. Furthermore, the subsequent analysis using Bayesian regression models provided interpretations regarding the associations between the wearer's attributes, such as disease type and gait ability, and the gait patterns of each subgroup. This study is the first to reveal the characteristics of entire gait kinematic and kinetic patterns during HAL-assisted walking in individuals with neuromuscular diseases, as well as to investigate how differences in gait patterns relate to disease type and gait ability. Our new insight into gait under the fusion of the wearer and the HAL will encourage the gait assessment using the data automatically measured by the HAL in cybernics treatment.

## 2 Materials and methods

### 2.1 Data source and study design

This study was a secondary analysis of data collected for a long-term outcome survey of “HAL for Medical Use (Lower Limb Type)” (Trial ID: jRCT1092220301/JMA-IIA00301) (24). One objective of the survey was to collect and analyze data on cybernics treatment using HAL. The participants in the survey were individuals with neuromuscular diseases, including the following conditions associated with gait disorder: amyotrophic lateral sclerosis, spinal muscular atrophy, spinal bulbar muscular atrophy, Charcot-Marie Tooth disease, distal myopathy, congenital myopathy, sporadic inclusion body myositis, and muscular dystrophy. The treatments were performed at 20 specialty care hospitals or clinics in Japan. Each intervention required participants to wear a HAL for Medical Use—Lower Limb Type (HAL-ML05) and walk under the fusion with the HAL for periods ranging between 20 and 40 min, with no more than one session conducted per day. The duration of the intervention was defined as a maximum of 40 months, and the number and duration of treatments differed among participants. As the primary outcome of the survey, the 2-min walk test was conducted without HAL at intervals.

The control mode of the HAL was set to Cybernic Voluntary Control (CVC) mode, in which the assisting torques to the hip and knee joint support the wearer's movement according to voluntary muscle activity (11, 25, 26). The degree of assist torques from the HAL was adjusted by the operators at each clinical site, depending on the wearer's condition (26). Gait data during treatment were automatically collected using the HAL and saved through dedicated applications. Time-series data of ground reaction forces (heel and toe), lower limb joint angles (hip and knee), trunk angles (pitch and roll), and joint torques of the HAL (hip and knee) were included. Gait time-series data, other than trunk angles, were obtained separately for each leg (left and right).

Gait data from 549 interventions in 48 individuals were obtained through the survey. From these data, the minimum number of interventions per person was 1, the maximum was 93, and the median was 6. The age of the participants at the start of the cybernics treatment ranged from 24 to 77 years. The distribution of height ranged from 149.8 cm to 179.0 cm, and regarding the sizes of HAL, 10 participants used size S, 28 used size M, 9 used size L, and 1 used size X.

### 2.2 Data processing

After obtaining the gait data measured by the HAL from the survey, we segmented the time-series data into gait cycles based on the weight distribution between the left and right legs calculated from the ground reaction forces (27). The start of the gait cycle was defined as the point at which a change in the weight distribution ratio occurred owing to a transition from the swing phase to ground contact (Supplementary Material A). The gait cycle starting with the initial contact of the right leg defined the right leg as the reference leg. Similarly, when the cycle started with the initial contact of the left leg, it was defined as the reference leg (Supplementary Material A). Subsequently, for each gait cycle, the time-series data were time-normalized in 101 time points, from 0% to 100% in 1% increments, using spline interpolation. The ground reaction force data were amplitude-normalized by dividing them by the wearer's body weight.

### 2.3 Principal component analysis

In the following analysis, we used gait time-series data from 15 gait cycles per treatment session for each leg side. The sessions in which the number of detected gait cycles did not reach 15 per leg were excluded from the analysis. Some clinical data included sessions with fewer gait cycles, and excluding these sessions could potentially introduce bias due to the wearer's gait ability and fatigue. The threshold of 15 cycles was determined by balancing the amount of excluded data with a sufficient amount of data to ensure consistency in the analysis. Ultimately, time-series data comprising 13,710 gait cycles from 457 treatments involving 48 individuals were used for the study.

We used principal component analysis (PCA) on the gait time-series data to summarize the major variations in gait patterns with low-dimensional features (28–32). A total of seven matrices for applying PCA were created for each set of kinematic and kinetic data. For the ground reaction forces, the data from the heel and toe sides of both the reference leg (the leg that begins bearing weight at 0% of the gait cycle) and the opposite leg were combined to form a matrix with 404 columns (101 time points × 4 items) and 13,710 rows (13,710 gait cycles). For the hip joint angle, the data for the reference leg were extracted from each gait cycle, resulting in a matrix of 101 columns (101 time points × 1 item). Similarly, matrices with 101 columns were created for the knee joint angle, hip torque, and knee torque. For the trunk pitch angle, the time-normalized data for each gait cycle were used directly to form a 101-column matrix. For the trunk roll angle, the signs were unified for the left and right legs such that the tilt toward the reference leg was in the positive direction, and a 101-column matrix was formed. All these matrices consisted of 13,710 rows. The eigenvectors obtained from the covariance matrix of each kinematic/kinetic data matrix served as the principal components (PCs) representing the independent features of the patterns. The PCs were ordered by the amount of captured variation in the time-series data, with the first PC corresponding to the greatest variation in the patterns of the data set (33). The principal component scores represent the contribution of each principal component to the time-series pattern of individual gait cycles (30).

### 2.4 Clustering analysis

We employed hierarchical clustering using Ward's linkage method and Euclidean distance measures (22, 23, 34–36) to determine the subgroups of each kinematic and kinetic gait pattern. In the cluster analysis of each measurement item, the input was the scores of the selected PCs up to the point where the cumulative contribution rate reached 99% in the PCA, ensuring that sufficient principal components were selected to avoid overly limiting the features influencing the clustering, while efficiently reducing dimensionality. The final number of clusters was set to that with the largest percentage change in linkage distance with the change in the number of clusters, out of an analytically tractable range of 5–10 (37).

### 2.5 Bayesian regression analysis

Our interest was to understand how the wearer's disease characteristics and gait ability were related to differences among the clustered subgroups. To address this critical issue in a clinical context, we constructed a Bayesian regression model for each kinematic and kinetic gait pattern to analyze the effects of disease type and 2-min walk distance on the distribution probabilities of the gait pattern subgroups in each treatment session (38).

Assuming that the wearer's disease type, the initial gait ability before the first treatment during the survey, and gait ability at each treatment session would make the difference in the gait patterns, we incorporated these variables into the regression model. The distribution of gait patterns was represented by the number of gait cycles belonging to each cluster in a single treatment session. A total of 30 gait cycles (15 cycles per leg) were extracted from each treatment session, so the response variable $y_{it}$ for the *t*-th session of participant *i* was modeled to follow a multinomial distribution with n=30, where the probability of belonging to *k*-th cluster was parameterized by $\theta_{itk}$. The parameter $\theta_{itk}$ was linked to a linear model through the softmax function. The linear predictor included the following variables: disease type $x_{1i}$, initial gait ability $x_{2i}$, and the gait ability at the *t*-th session $x_{3it}$ for participant *i*. Additionally, a random effect $u_{ik}$ was included in the model to account for individual differences, as the data were collected repeatedly from each participant.

Regarding disease type ($x_{1i}$), the eight neuromuscular diseases were divided into two groups: neurogenic disease, including amyotrophic lateral sclerosis, spinal muscular atrophy, spinal bulbar muscular atrophy, and Charcot-Marie Tooth disease (coded as “0” in the binary variable); and myogenic disease, including distal myopathy, congenital myopathy, sporadic inclusion body myositis, and muscular dystrophy (coded as “1”). For initial gait ability ($x_{2i}$), we used the 2-min walk distance measured before each participant's first treatment session. Gait ability at the *t*-th treatment session ($x_{3it}$) was represented by the deviation from each participant's average 2-min walk distance. Since 2-min walk distances were not measured at every treatment session but before and after one series of several sessions, the 2-min walk distances for each session within a series were estimated using linear interpolation. If either the pre- or post-series measurement was missing, the available measurement was applied for all sessions within the series. In cases where both measurements were missing, the data from that series were excluded as missing data. Ultimately, data from 428 treatment sessions across 45 participants were used in this Bayesian analysis.

The Bayesian regression model described above is expressed by the following equation:

$$\begin{array}{rl} & y_{it}∼\mathrm{Multinomial}(n,\theta_{it}),\\ & \theta_{it}=(\theta_{it1},\;\theta_{it2},\;\ldots ,\;\theta_{itK}),\\ & \theta_{itk}=\frac{\exp(\eta_{itk})}{\sum_{\;j=1}^{K}\exp(\eta_{itj})},\\ & \eta_{itk}=\beta_{0k}+\beta_{1k}x_{1i}+\beta_{2k}x_{2i}+\beta_{3k}x_{3it}+u_{ik}, \end{array}$$

where *K* is the number of subgrouped clusters (k=1,2,…,K) for each measurement item. All parameters were allowed to take both positive and negative real values, and we used a normal distribution with a mean of 0 as the prior distribution (38). To account for the differences in scale between 2-min walk distances and other variables, the variances were set to 1 for *β*_0*k*_, *β*_1*k*_, and $u_{ik}$, and to 0.01 for *β*_2*k*_ and *β*_3*k*_:

$$\begin{array}{rl} & \beta_{0k},\;\beta_{1k},\;u_{ik}∼N(0,\;1),\\ & \beta_{2k},\;\beta_{3k}∼N(0,\;0.01).\; \end{array}$$

The impacts of explanatory variables on the distribution probability of gait patterns were interpreted based on the sign of each parameter. We considered a parameter to have a strong effect if the 95% credible interval (95% CI) of its posterior distribution did not include zero (39).

The Bayesian regression analysis was conducted using the PyMC package (v5.12.0) in Python (40). The posterior distributions were estimated using the No-U-Turn Sampler (NUTS) (41). The sampling was performed using four chains with 4,000 iterations each, with the first 2,000 iterations discarded as the burn-in period. Convergences were assessed by the Gelman-Rubin statistic ($\hat{R}$) (38), which was 1.0 for all parameters.

## 3 Results

### 3.1 Ground reaction forces

**Figure 2:**
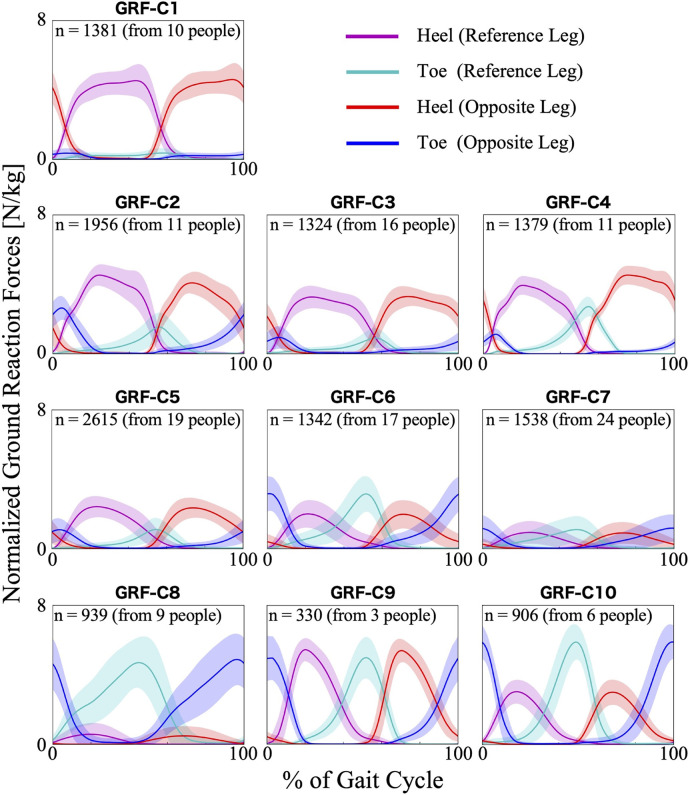
Patterns of ground reaction forces for each subgroup. The loads are normalized by the wearer's weight. The *n* in the graph is the number of gait cycles in the cluster. The solid lines are the average patterns of all gait cycles in the cluster, while the colored shadings are the mean ± standard deviation range. The magenta and cyan colors represent the values measured by the sensors on the heel and toe side of the reference leg (the leg that begins bearing weight at 0% of the gait cycle), respectively. The red and blue colors represent the heel and toe side of the opposite leg, respectively.

The first 23 PCs, accounting for a cumulative contribution rate of 99.02%, were selected as clustering inputs. The patterns of the ground reaction forces were divided into ten subgroups (GRF-C1–C10; Figure 2) at a percentage change in the linkage distance of 21.92%. Figure 2 shows that the load on the heel side was greater than that on the toe side for GRF-C1–C5. Conversely, for C6, 8, and 10, the load on the toe side was greater than that on the heel side. In C1 and C8 the load was primarily on one side, either the heel side or the toe side, respectively. In the GRF-C7 pattern, the peak loads of the normalized ground reaction forces were smaller than those of the other clusters. In contrast, C9 has large loads on both the heel and toe sides. There was a left-right difference in C2 and C4, where the heel load of one leg and the toe load of the other leg were greater as a set. The results of the Bayesian analysis (Table 1) indicated that disease type (*β*_1*k*_) had a positive effect on GRF-C4 and negative effects on C1 and C9, meaning that myogenic diseases increased the likelihood of C4 and decreased it for C1 and C9 compared to neurogenic diseases. Initial gait ability (*β*_2*k*_) tended to have negative effects on GRF-C3 and C7, and positive effects on C9 and C10. Gait ability at each treatment session (*β*_3*k*_) positively influenced GRF-C1 and C6, and negatively influenced C4 and C7. The baseline probability of cluster membership, excluding the effects of disease type and gait ability, was higher for GRF-C1, C3, C5, and C7, with positive 95% CIs for the intercept (*β*_0*k*_), and lower for C4, C9, and C10, with negative 95% CIs for the intercept.

**Table 1:** Posterior summary and convergence statistics of the Bayesian regression model for ground reaction forces.

	Intercept (*β*_0*k*_)			Disease type (*β*_1*k*_)			Initial gait ability (*β*_2*k*_)			Session gait ability (*β*_3*k*_)		
	Mean	[95% CI]	ESS	Mean	[95% CI]	ESS	Mean	[95% CI]	ESS	Mean	[95% CI]	ESS
C1	1.025	[0.049, 2.025]	4,006	−1.484	[−2.391, −0.493]	4,330	−0.002	[−0.012, 0.007]	4,849	0.016	[0.004, 0.028]	8,385
C2	−0.381	[−1.428, 0.619]	4,838	0.428	[−0.543, 1.305]	4,331	0.002	[−0.007, 0.012]	4,907	−0.009	[−0.019, 0.001]	6,314
C3	1.105	[0.188, 2.079]	4,098	0.568	[−0.288, 1.534]	3,860	−0.012	[−0.021, −0.002]	4,878	−0.004	[−0.013, 0.005]	5,300
C4	−1.393	[−2.506, −0.308]	5,018	1.035	[0.042, 2.005]	4,669	0.001	[−0.01, 0.011]	5,344	−0.029	[−0.039, −0.019]	5,770
C5	1.213	[0.335, 2.224]	3,475	0.805	[−0.054, 1.679]	3,422	−0.004	[−0.013, 0.005]	4,229	−0.004	[−0.013, 0.004]	4,348
C6	0.633	[−0.300, 1.539]	3,926	0.369	[−0.492, 1.226]	3,519	0.004	[−0.005, 0.013]	4,174	0.031	[0.021, 0.042]	6,604
C7	3.134	[2.239, 4.029]	3,418	0.553	[−0.331, 1.418]	3,610	−0.029	[−0.038, −0.019]	4,678	−0.022	[−0.031, −0.012]	5,541
C8	0.829	[−0.125, 1.785]	4,147	0.103	[−0.855, 1.022]	4,335	−0.01	[−0.019, 0.000]	5,045	−0.007	[−0.021, 0.008]	10,648
C9	−2.206	[−3.428, −0.995]	6,401	−2.33	[−3.565, −1.106]	6,516	0.017	[0.006, 0.028]	5,173	0.017	[0.000, 0.033]	11,376
C10	−3.814	[−5.038, −2.601]	6,144	−0.119	[−1.160, 0.911]	4,979	0.031	[0.021, 0.042]	4,937	0.009	[−0.008, 0.024]	11,361

The table presents the mean and 95% credible intervals (95% CI) of the posterior distribution for fixed effects, as well as the bulk effective sample size (ESS) for each parameter. The rows correspond to the cluster numbers (*k*) of subgrouped patterns; i.e., C*k* corresponds to the cluster name “GRF-C*k*”.

### 3.2 Lower limb joint angles

**Figure 3:**
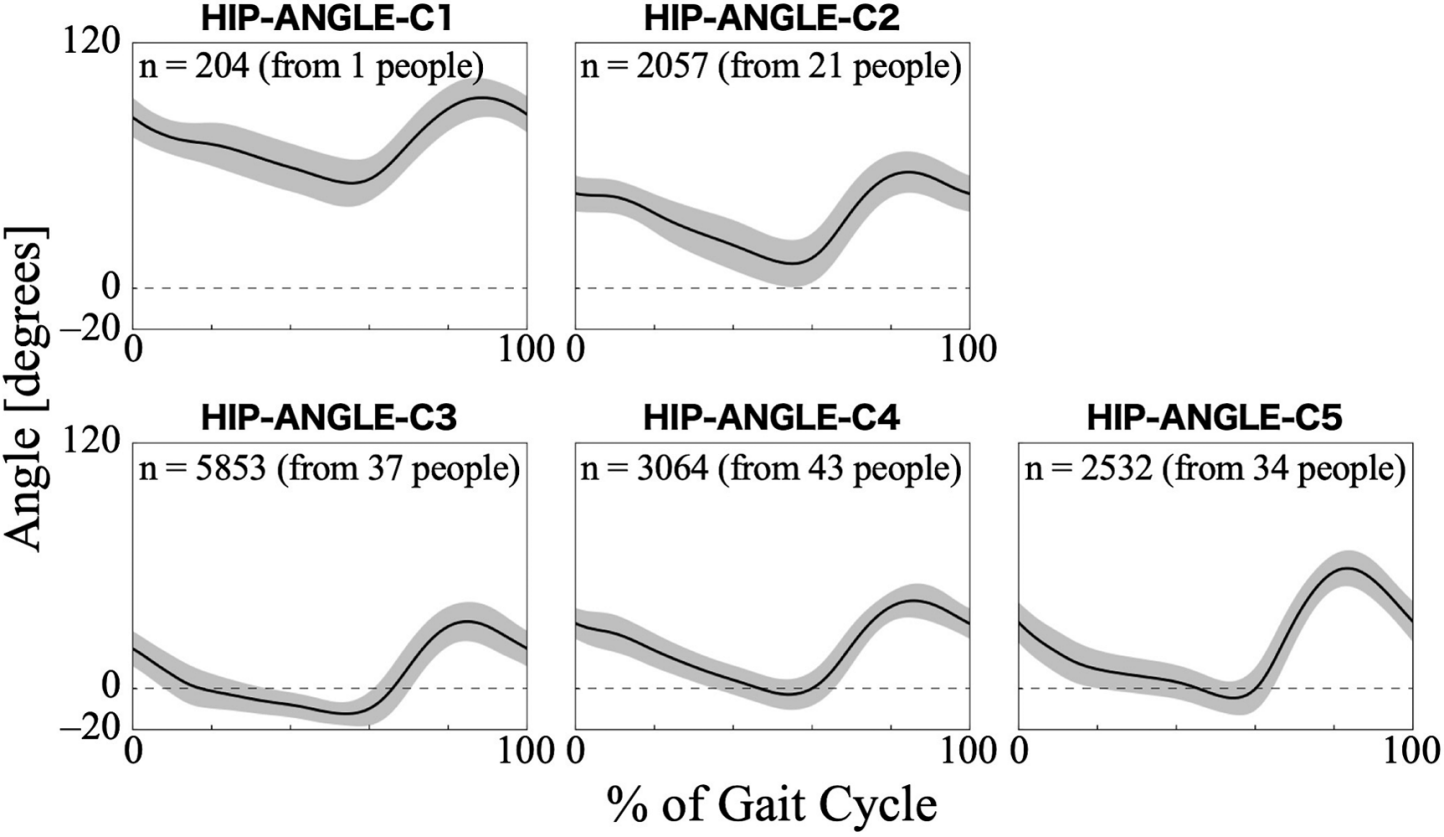
Patterns of hip joint angle for each subgroup. Positive angles indicate flexion, negative angles indicate extension, and zero indicates the hip joint is straight. The *n* in the graph is the number of gait cycles in the cluster. The solid black line is the average pattern of all gait cycles in the cluster, and the gray shading is the mean ± standard deviation range.

For the hip joint angle, the first eight PCs, which accounted for a cumulative contribution rate of 99.07%, were selected as clustering inputs. The hip angle patterns were divided into five subgroups (HIP-ANGLE-C1–C5; Figure 3) at a percentage change in the linkage distance of 12.04%. Figure 3 shows that the primary differences in the hip joint angle patterns were in the magnitude of the offset throughout the gait cycle and the range of motion. HIP-ANGLE-C1, which was composed solely of data from one participant, had the largest flexion direction offset over the entire gait cycle. C2 also showed a strong tendency toward flexion, although not as much as C1, and its average pattern did not extend below 0°. In cluster C3, the timing to reach extension was the earliest, and the maximum extension angle was the largest among the five clusters. The patterns included in C5 indicated a greater range of hip-angle motion with large flexion in the swing phase. The posterior distribution from the Bayesian analysis (Table 2) indicated that initial gait ability (*β*_2*k*_) had a negative effect on HIP-ANGLE-C1 and a positive effect on C3. The baseline probability of cluster membership was lower for C1, with a negative 95% CI for the intercept (*β*_0*k*_), and higher for C4 and C5, with positive 95% CIs for the intercept.

**Table 2:** Posterior summary and convergence statistics of the Bayesian regression model for hip joint angle.

	Intercept (*β*_0*k*_)			Disease type (*β*_1*k*_)			Initial gait ability (*β*_2*k*_)			Session gait ability (*β*_3*k*_)		
	Mean	[95% CI]	ESS	Mean	[95% CI]	ESS	Mean	[95% CI]	ESS	Mean	[95% CI]	ESS
C1	−2.740	[−3.947, −1.490]	9,853	0.261	[−1.087, 1.512]	10,649	−0.023	[−0.038, −0.008]	11,347	−0.001	[−0.019, 0.017]	16,278
C2	−0.237	[−1.259, 0.847]	7,160	0.721	[−0.320, 1.756]	6,957	−0.003	[−0.013, 0.008]	8,715	0.008	[−0.002, 0.017]	6,428
C3	0.522	[−0.500, 1.521]	6,893	−0.388	[−1.420, 0.612]	6,858	0.016	[0.006, 0.027]	8,506	−0.008	[−0.018, 0.002]	6,755
C4	1.126	[0.105, 2.129]	6,484	0.156	[−0.870, 1.150]	7,100	0.008	[−0.002, 0.019]	7,666	−0.001	[−0.010, 0.010]	6,313
C5	1.288	[0.250, 2.343]	7,050	−0.730	[−1.761, 0.261]	7,370	0.002	[−0.008, 0.013]	8,331	0.002	[−0.008, 0.013]	6,395

The table presents the mean and 95% credible intervals (95% CI) of the posterior distribution for fixed effects, as well as the bulk effective sample size (ESS) for each parameter. The rows correspond to the cluster numbers (*k*) of subgrouped patterns; i.e., C*k* corresponds to the cluster name “HIP-ANGLE-C*k*”.

**Figure 4:**
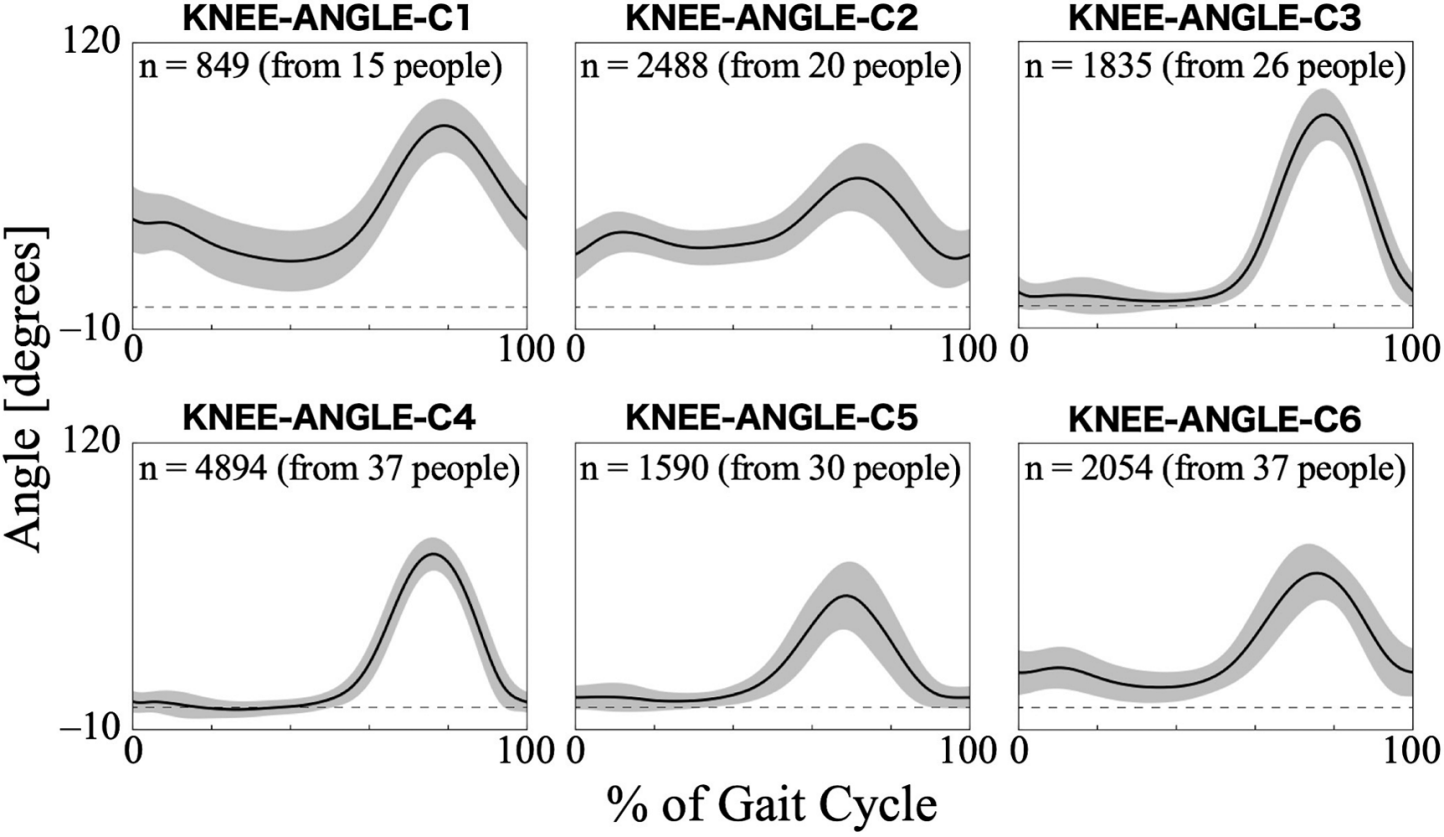
Patterns of knee joint angle for each subgroup. Positive angles indicate flexion, negative angles indicate extension, and zero indicates the knee joint is straight. The *n* in the graph is the number of gait cycles in the cluster. The solid black line is the average pattern of all gait cycles in the cluster, and the gray shading is the mean ± standard deviation range.

For the knee joint angle, the first nine PCs, which accounted for a cumulative contribution rate of 99.26%, were selected as clustering inputs. The knee angle patterns were divided into six subgroups (KNEE-ANGLE-C1–C6; Figure 4), with a percentage change in the linkage distance of 17.24%. Figure 4 shows that KNEE-ANGLE-C1 and C2 had weak stance-phase extensions, whereas C3–C5 reached a knee joint angle near 0° in the stance phase. The maximum flexion angles were larger in C1 and C3 than in the other clusters, whereas it was smaller in C5. The posterior distribution from the Bayesian analysis (Table 3) showed a trend of fewer gait cycles in KNEE-ANGLE-C3 and more in C5 with myogenic diseases compared to neurogenic diseases (*β*_1*k*_). Both lower initial gait ability (*β*_2*k*_) and lower session gait ability (*β*_3*k*_) were more likely to produce patterns in KNEE-ANGLE-C2. The baseline probability of cluster membership was lower for C1, with a negative 95% CI for the intercept (*β*_0*k*_).

**Table 3:** Posterior summary and convergence statistics of the Bayesian regression model for knee joint angle.

	Intercept (*β*_0*k*_)			Disease type (*β*_1*k*_)			Initial gait ability (*β*_2*k*_)			Session gait ability (*β*_3*k*_)		
	Mean	[95% CI]	ESS	Mean	[95% CI]	ESS	Mean	[95% CI]	ESS	Mean	[95% CI]	ESS
C1	−1.151	[−2.161, −0.071]	3,695	−0.320	[−1.294, 0.675]	3,388	−0.002	[−0.012, 0.008]	3,952	−0.012	[−0.023, 0.000]	6,370
C2	0.330	[−0.688, 1.301]	3,229	0.395	[−0.583, 1.355]	3,145	−0.011	[−0.021, −0.002]	3,530	−0.013	[−0.022, −0.004]	3,836
C3	−0.334	[−1.270, 0.661]	3,422	−0.974	[−1.964, −0.026]	3,043	0.008	[−0.002, 0.017]	3,314	0.009	[−0.001, 0.019]	4,269
C4	0.717	[−0.252, 1.651]	3,021	−0.337	[−1.299, 0.570]	2,862	0.007	[−0.002, 0.016]	3,476	0.007	[−0.001, 0.016]	3,456
C5	−0.293	[−1.249, 0.655]	3,247	1.287	[0.354, 2.257]	2,981	−0.003	[−0.013, 0.006]	3,443	0.000	[−0.009, 0.009]	3,836
C6	0.693	[−0.264, 1.630]	3,025	−0.052	[−0.984, 0.881]	2,938	0.002	[−0.008, 0.011]	3,111	0.008	[−0.001, 0.016]	3,408

The table presents the mean and 95% credible intervals (95% CI) of the posterior distribution for fixed effects, as well as the bulk effective sample size (ESS) for each parameter. The rows correspond to the cluster numbers (*k*) of subgrouped patterns; i.e., C*k* corresponds to the cluster name “KNEE-ANGLE-C*k*”.

### 3.3 Trunk angles

**Figure 5:**
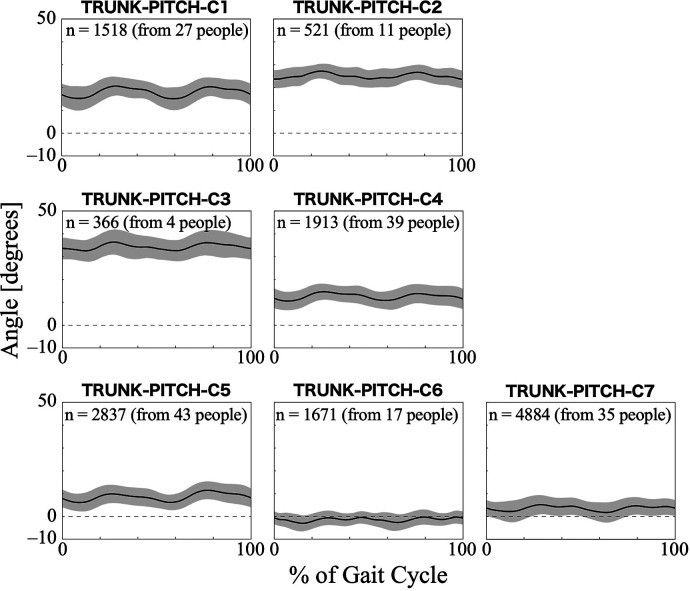
Patterns of trunk pitch angle for each subgroup. The trunk pitch angle represents the tilt of the HAL control system behind the wearer's lumbar at the sagittal plane. Positive trunk pitch angles indicate anterior tilt, negative trunk pitch angles indicate posterior tilt, and zero indicates upright posture with no tilt. The *n* in the graph is the number of gait cycles in the cluster. The solid black line is the average pattern of all gait cycles in the cluster, and the gray shading is the mean ± standard deviation range.

For the trunk pitch angle, the first 11 PCs, which accounted for a cumulative contribution rate of 99.18%, were selected as clustering inputs. The trunk pitch angle patterns were divided into seven subgroups (TRUNK-PITCH-C1–C7; Figure 5) with a 33.60% change in the linkage distance. Figure 5 shows that the pattern of the trunk pitch angles can be divided by the degree of forward tilt. The magnitude of the anterior tilt of the trunk was not in the order of the cluster number, with TRUNK-ROLL-C3 having the largest forward lean angle. On the contrary, in C6, the average pattern of the pitch angle remained in the negative range throughout the gait cycle, indicating a slight backward lean. The results of the Bayesian analysis (Table 4) suggested that having myogenic rather than neurogenic diseases (*β*_1*k*_) increased the likelihood of the pattern in TRUNK-PITCH-C2 and C3, and decreased the likelihood of C5. Initial gait ability (*β*_2*k*_) tended to have negative effects on TRUNK-PITCH-C2 and C3, and positive effects on C5–7. Gait ability at each treatment session (*β*_3*k*_) positively influenced TRUNK-PITCH-C3, and negatively influenced C1 and C6. The baseline probability of cluster membership was higher for TRUNK-PITCH-C1, C4, and C5, with positive 95% CIs for the intercept (*β*_0*k*_), and lower for C3 and C6, with negative 95% CIs for the intercept.

**Table 4:** Posterior summary and convergence statistics of the Bayesian regression model for trunk pitch angle.

	Intercept (*β*_0*k*_)			Disease type (*β*_1*k*_)			Initial gait ability (*β*_2*k*_)			Session gait ability (*β*_3*k*_)		
	Mean	[95% CI]	ESS	Mean	[95% CI]	ESS	Mean	[95% CI]	ESS	Mean	[95% CI]	ESS
C1	0.96	[0.028, 1.875]	7,129	−0.595	[−1.512, 0.342]	6,610	0.001	[−0.008, 0.011]	8,394	−0.018	[−0.026, −0.009]	7,266
C2	−0.991	[−2.039, 0.099]	9,231	1.719	[0.598, 2.823]	8,967	−0.026	[−0.039, −0.014]	10,821	0.003	[−0.007, 0.012]	7,974
C3	−1.893	[−3.113, −0.751]	11,272	1.460	[0.192, 2.639]	10,677	−0.029	[−0.042, −0.014]	13,106	0.036	[0.023, 0.049]	14,460
C4	1.169	[0.201, 2.048]	6,995	−0.774	[−1.674, 0.181]	5,963	0.009	[0.000, 0.019]	8,239	−0.002	[−0.011, 0.006]	6,543
C5	1.272	[0.376, 2.214]	6,368	−0.932	[−1.842, −0.045]	6,557	0.016	[0.007, 0.025]	8,131	0.003	[−0.006, 0.011]	6,670
C6	−1.096	[−2.064, −0.070]	8,140	−0.349	[−1.315, 0.639]	7,391	0.011	[0.001, 0.021]	9,114	−0.017	[−0.026, −0.008]	7,507
C7	0.637	[−0.295, 1.562]	7,044	−0.572	[−1.512, 0.342]	6,081	0.018	[0.008, 0.027]	8,426	−0.004	[−0.012, 0.004]	7,074

The table presents the mean and 95% credible intervals (95% CI) of the posterior distribution for fixed effects, as well as the bulk effective sample size (ESS) for each parameter. The rows correspond to the cluster numbers (*k*) of subgrouped patterns; i.e., C*k* corresponds to the cluster name “TRUNK-PITCH-C*k*”.

**Figure 6:**
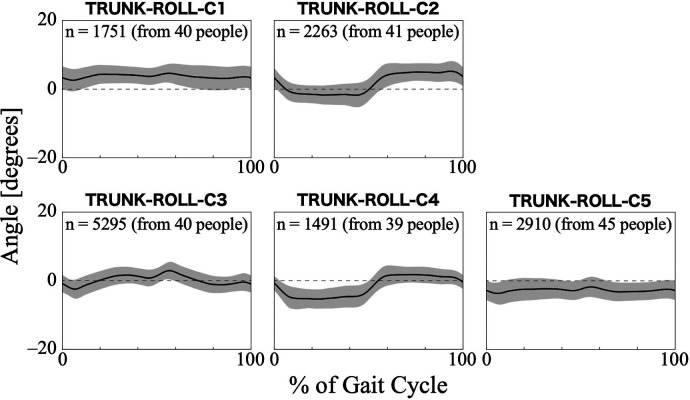
Patterns of trunk roll angle for each subgroup. The trunk roll angle represents the tilt of the HAL control system behind the wearer's lumbar at the frontal plane. Positive trunk roll angles indicate tilt toward the reference leg side, negative trunk roll angles indicate tilt toward the opposite leg side, and zero indicates upright posture with no tilt. The *n* in the graph is the number of gait cycles in the cluster. The solid black line is the average pattern of all gait cycles in the cluster, and the gray shading is the mean ± standard deviation range.

For the trunk roll angle, the first 13 PCs, which accounted for a cumulative contribution rate of 99.07%, were selected as clustering inputs. The trunk roll angle patterns were divided into five subgroups (TRUNK-ROLL-C1–C5; Figure 6) with a percentage change in the linkage distance of 38.83%. Figure 6 shows that the roll angle of the trunk varies with the symmetry and magnitude of the trunk sway. The lateral sway of the trunk was small throughout the gait cycle in TRUNK-ROLL-C1 and C5, and both clusters showed left-right differences. In C1, the trunk generally leaned toward the side of the reference leg, making an initial contact. In contrast, in C5, the trunk leaned toward the side of the non-reference leg. The patterns in C2 and C4 had common characteristics in that there were large changes in the angles at the beginning (approximately 0%–10%) and middle (approximately 50%–60%) of the gait cycle, with the trunk leaning toward the opposite non-reference leg in the first half and toward the side of the reference leg in the second half. However, they differed in whether the leaning angles were greater for the reference leg or the opposite leg. C3 exhibited a symmetrical pattern where the trunk reached a peak tilt near 0%–10% and 50%–60% of the gait cycle, followed quickly by a return to vertical orientation. The results of the Bayesian analysis (Table 5) suggested that being neurogenic rather than myogenic (*β*_1*k*_) increased the likelihood of the pattern in TRUNK-ROLL-C4 and that lower gait ability at treatment sessions (*β*_3*k*_) increased the likelihood of the pattern in C3.

**Table 5:** Posterior summary and convergence statistics of the Bayesian regression model for trunk roll angle.

	Intercept (*β*_0*k*_)			Disease TYPE (*β*_1*k*_)			Initial gait ability (*β*_2*k*_)			Session gait ability (*β*_3*k*_)		
	Mean	[95% CI]	ESS	Mean	[95% CI]	ESS	Mean	[95% CI]	ESS	Mean	[95% CI]	ESS
C1	−0.496	[−1.538, 0.467]	8,100	0.531	[−0.436, 1.524]	7,680	−0.003	[−0.013, 0.007]	9,284	−0.002	[−0.012, 0.007]	7,819
C2	0.064	[−0.956, 1.059]	8,330	−1.03	[−1.991, 0.015]	7,608	0.007	[−0.002, 0.017]	8,352	0.008	[−0.002, 0.017]	8,380
C3	0.316	[−0.653, 1.338]	8,114	0.925	[−0.047, 1.961]	7,579	−0.004	[−0.013, 0.006]	9,009	−0.012	[−0.021, −0.003]	8,061
C4	0.143	[−0.874, 1.134]	8,203	−1.152	[−2.155, −0.164]	7,607	0.001	[−0.010, 0.010]	9,060	0.009	[0.000, 0.019]	8,141
C5	−0.033	[−1.016, 1.005]	7,977	0.747	[−0.220, 1.743]	7,472	−0.002	[−0.012, 0.008]	9,179	−0.002	[−0.012, 0.006]	8,078

The table presents the mean and 95% credible intervals (95% CI) of the posterior distribution for fixed effects, as well as the bulk effective sample size (ESS) for each parameter. The rows correspond to the cluster numbers (*k*) of subgrouped patterns; i.e., C*k* corresponds to the cluster name “TRUNK-ROLL-C*k*”.

### 3.4 Joint torques of HAL

**Figure 7:**
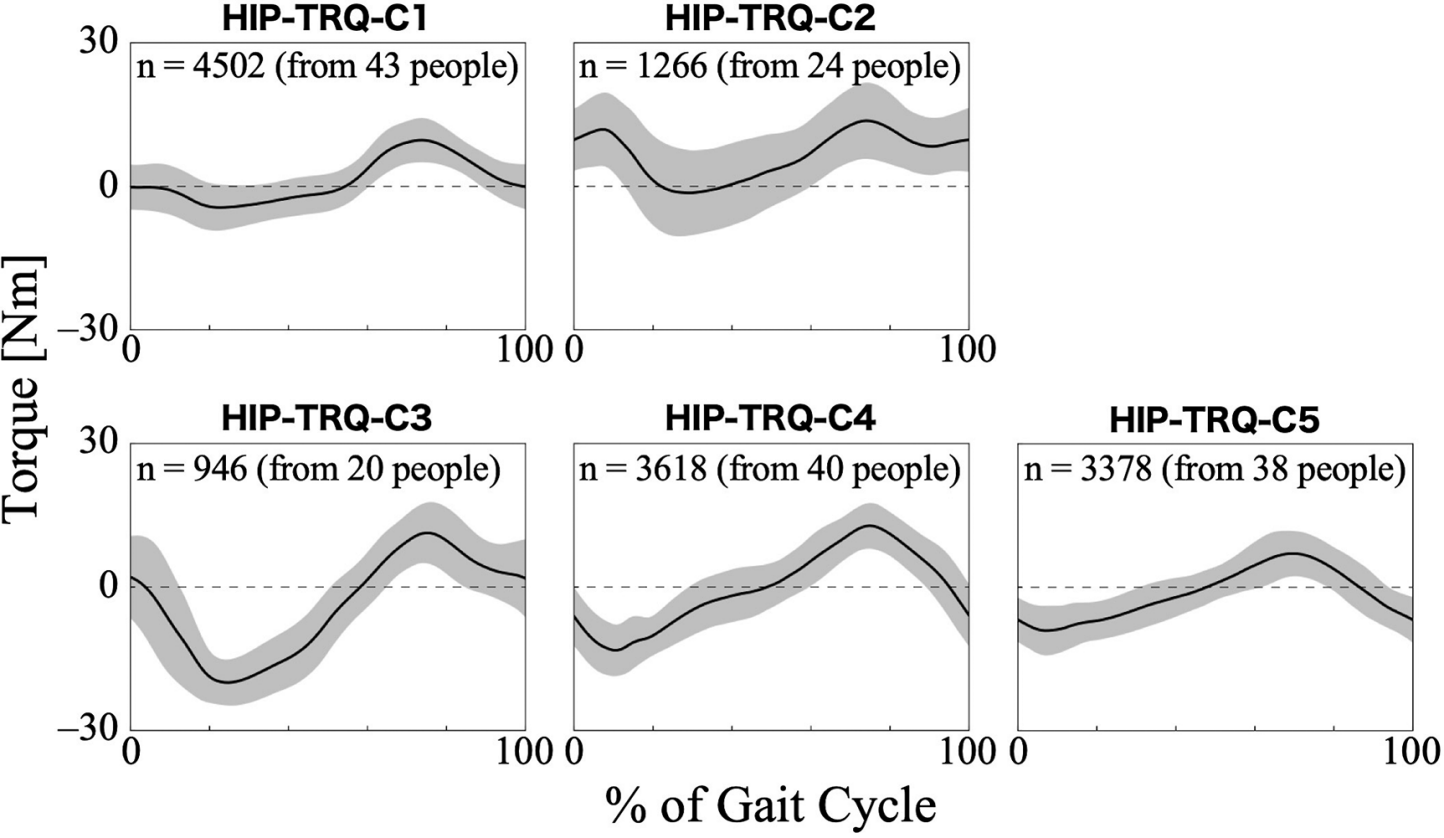
Patterns of hip joint torque produced by HAL for each subgroup. Positive torque indicates a force in the flexion direction, negative torque indicates in the extension direction, and zero indicates no torque is applied. The *n* in the graph is the number of gait cycles in the cluster. The solid black line is the average pattern of all gait cycles in the cluster, and the gray shading is the mean ± standard deviation range.

For the torque of the HAL at the hip joint, the first 22 PCs, accounting for a cumulative contribution rate of 99.11%, were selected as clustering inputs. The patterns of the HAL's hip torque were divided into five subgroups (HIP-TRQ-C1–C5; Figure 7), with a percentage change in the linkage distance of 35.40%. Figure 7 shows that there were differences in the patterns of the HAL hip joint torque during the first half of the gait cycle. During this phase, HIP-TRQ-C3, C4, and C5 showed clear extension torque, whereas C1 showed only a slight extension tendency. Unlike the other clusters, C2 initially showed flexion torque and had a strong flexion tendency throughout the gait cycle. In C3, the peak extension torque occurred later than in C4 and C5. The difference between C4 and C5 was the torque amplitude. The posterior distribution from the Bayesian analysis (Table 6) showed a trend toward more gait cycles in HIP-TRQ-C4 for neurogenic diseases, with a negative effect of disease type (*β*_1*k*_). Initial gait ability (*β*_2*k*_) tended to have a negative effect on C2 and a positive effect on C5. Gait ability at each treatment session (*β*_3*k*_) negatively influenced C1 and positively influenced C2. The baseline probability of cluster membership was lower for HIP-TRQ-C3, with a negative 95% CI for the intercept (*β*_0*k*_), and higher for C4, with a positive 95% CI for the intercept.

**Table 6:** Posterior summary and convergence statistics of the Bayesian regression model for hip joint torque produced by HAL.

	Intercept (*β*_0*k*_)			Disease type (*β*_1*k*_)			Initial gait ability (*β*_2*k*_)			Session gait ability (*β*_3*k*_)		
	Mean	[95% CI]	ESS	Mean	[95% CI]	ESS	Mean	[95% CI]	ESS	Mean	[95% CI]	ESS
C1	0.505	[−0.534, 1.497]	8,619	0.525	[−0.488, 1.499]	8222	0.009	[−0.001, 0.019]	10,488	−0.013	[−0.022, −0.004]	8,537
C2	−0.289	[−1.325, 0.749]	9,428	0.601	[−0.451, 1.611]	9,069	−0.011	[−0.021, −0.001]	10,749	0.017	[0.007, 0.026]	8,143
C3	−1.049	[−2.130, −0.011]	9,484	−0.128	[−1.214, 0.894]	9,073	−0.011	[−0.022, 0.000]	12,271	0.002	[−0.008, 0.012]	8,163
C4	1.539	[0.554, 2.580]	8,931	−1.373	[−2.340, −0.373]	8,297	−0.001	[−0.012, 0.009]	10,725	−0.005	[−0.014, 0.005]	7,954
C5	−0.744	[−1.790, 0.242]	8,794	0.438	[−0.519, 1.475]	8,574	0.014	[0.004, 0.024]	10,203	−0.001	[−0.011, 0.008]	8,188

The table presents the mean and 95% credible intervals (95% CI) of the posterior distribution for fixed effects, as well as the bulk effective sample size (ESS) for each parameter. The rows correspond to the cluster numbers (*k*) of subgrouped patterns; i.e., C*k* corresponds to the cluster name “HIP-TRQ-C*k*”.

**Figure 8:**
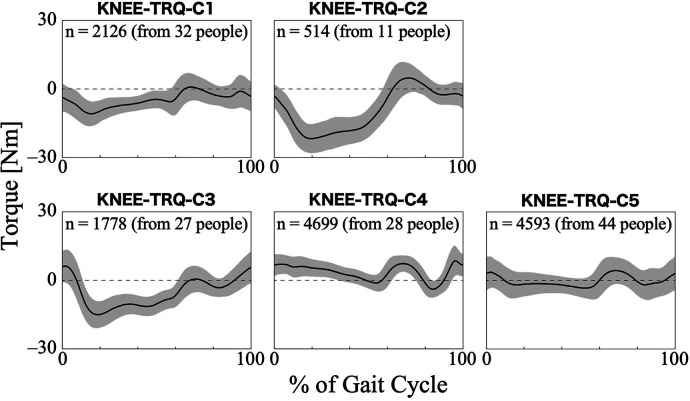
Patterns of knee joint torque produced by HAL for each subgroup. Positive torque indicates a force in the flexion direction, negative torque indicates in the extension direction, and zero indicates no torque is applied. The *k* in the graph is the number of gait cycles in the cluster. The solid black line is the average pattern of all gait cycles in the cluster, and the gray shading is the mean ± standard deviation range.

For the torque of the HAL at the knee joint, the first 26 PCs, accounting for a cumulative contribution rate of 99.06%, were selected as clustering inputs. The patterns of the HAL's knee torque were divided into five subgroups (KNEE-TRQ-C1–C5; Figure 8), with a percentage change in the linkage distance of 17.64%. Figure 8 shows that there were differences among the clusters in the pattern of the HAL knee joint torque up to approximately 60% of the gait cycle. KNEE-TRQ-C1 to C3 mainly had extension torque during that phase, while C4 had flexion torque, and C5 had intermediate torque. In C2 and C3, the change in the torque values in the extension direction was large, up to approximately 20% of the gait cycle. The posterior distribution from the Bayesian analysis (Table 7) showed that those with lower initial gait ability (*β*_2*k*_) were more likely to take the KNEE-TRQ-C2 pattern. Lower gait ability at treatment sessions tended to increase the likelihood of C2, while higher gait ability at treatment sessions tended to increase the likelihood of C3 (*β*_3*k*_). The baseline probability was lower for KNEE-TRQ-C2 and C3, with negative 95% CIs for the intercept (*β*_0*k*_), and higher for C5, with a positive 95% CI for the intercept.

**Table 7:** Posterior summary and convergence statistics of the Bayesian regression model for knee joint torque produced by HAL.

	Intercept (*β*_0*k*_)			Disease type (*β*_1*k*_)			Initial gait ability (*β*_2*k*_)			Session gait ability (*β*_3*k*_)		
	Mean	[95% CI]	ESS	Mean	[95% CI]	ESS	Mean	[95% CI]	ESS	Mean	[95% CI]	ESS
C1	0.68	[−0.328, 1.777]	4,306	0.524	[−0.425, 1.539]	4,106	−0.005	[−0.016, 0.004]	4,763	0.008	[−0.001, 0.018]	4,190
C2	−1.502	[−2.633, −0.369]	4,665	0.966	[−0.173, 2.073]	4,998	−0.016	[−0.028, −0.005]	6,018	−0.029	[−0.040, −0.017]	6,040
C3	−1.364	[−2.401, −0.245]	4,679	0.142	[−0.901, 1.134]	4,328	0.007	[−0.003, 0.017]	4,918	0.011	[0.002, 0.021]	4,448
C4	0.233	[−0.825, 1.252]	3,825	−0.837	[−1.815, 0.205]	4,060	0.008	[−0.003, 0.018]	4,622	0.006	[−0.004, 0.016]	4,207
C5	1.964	[0.933, 2.969]	4,001	−0.833	[−1.816, 0.171]	4,097	0.006	[−0.003, 0.017]	4,600	0.003	[−0.006, 0.013]	4,016

The table presents the mean and 95% credible intervals (95% CI) of the posterior distribution for fixed effects, as well as the bulk effective sample size (ESS) for each parameter. The rows correspond to the cluster numbers (*k*) of subgrouped patterns; i.e., C*k* corresponds to the cluster name “KNEE-TRQ-C*k*”.

## 4 Discussion

The main objective of this study was to create subgroups of gait patterns that the HAL automatically measures during HAL-assisted gait in individuals with neuromuscular diseases. We hypothesized that the gait produced by the interaction between the wearer and HAL based on voluntary intention has various patterns. By forming subgroups using hierarchical clustering, we expected it would be possible to find typical gait types. The secondary goal was to clarify the characteristics of each cluster, particularly regarding the wearer's attributes that influence differences in gait patterns. We hypothesized that disease type and gait ability would affect the differences between clusters and expected that the Bayesian regression model would be useful in interpreting these effects. The overall results showed that the subgroups organized based on hierarchical clustering highlighted the characteristics of multiple kinematic and kinetic gait patterns while wearing HAL, with some patterns showing significant associations with the wearer's disease type and gait ability.

For the ground reaction forces, it is an obvious feature whether the load is biased toward the heel or toe. In a typical gait in individuals without gait disorders and without HAL, during the stance phase, the foot contacts the ground from the heel side, followed by a gradual forward shift of the body's center of mass, and finally, the toes leave the ground (42). The vertical component of the ground reaction force across the entire sole forms an M-shaped curve, with the first peak occurring soon after the heel strike and the second peak during push-off (42, 43). When wearing the HAL, because the ground reaction force sensors are positioned at the forward and backward of the sole, it was presumed that the first peak of the M-shaped curve was observed at the heel side and the second peak at the toe side. However, some data in the results showed load distributions that were different from the typical HAL non-wearing gait, as seen in several clusters represented by GRF-C1 and C8 (Figure 2). Generally, muscle weakness is thought to be a cause of heel walking (44–46), and it is possible that muscle weakness associated with neuromuscular diseases similarly leads to a lack or delay in heel-off during walking with the HAL. According to the Bayesian regression, the heel walking pattern of almost no load on the toes, as seen in GRF-C1, appears to be more likely in wearers with neurogenic diseases than in those with myogenic diseases. On the other hand, even among individuals with neurogenic diseases, those with higher initial gait ability tend to have an increased likelihood of the GRF-C9 or C10 patterns, where more stress is placed on the toes, suggesting that gait patterns vary significantly with disease progression. Toe walking is caused by spasticity or contracture of the plantar flexor muscles (44, 45). However, these are not commonly observed in individuals with neuromuscular diseases who participated in the outcome survey. Therefore, there may be specific contributing factors related to gait during the HAL intervention. Although the possibility of differences in ground reaction force patterns due to changes in gait speed and the presence or absence of HAL needs to be considered, the diversity in the ratio of load distribution between the heel and toe, as seen in the clustering results (Figure 2), would be a distinguishing and characteristic of these patterns.

A possible reason for the smaller loads of the normalized ground reaction forces observed in the patterns within GRF-C7 (Figure 2) is that the wearers used partial body weight support using gait harnesses during these interventions. Clusters of ground reaction forces other than GRF-C7 may also contain gait cycles in which weight support is used. Clinical operators sometimes combine HAL assistance and weight support, depending on the wearer's gait ability. If the wearer has difficulty supporting their own body weight while walking, clinical operators may increase the amount of body weight support. The results of the Bayesian regression, showing that both initial and session gait ability had a negative effect on GRF-C7, support the idea that a decline in gait ability increases the amount of weight support and reduces ground reaction forces throughout the gait cycle. The amplitude of the normalized ground reaction forces can be one of the key indicators of gait changes during intervention because it reflects the amount of weight support.

One of the characteristics that divided the hip joint angle patterns was the difference in the range of motion. Considering that the range of motion in natural speed gait while not wearing the HAL spans from 10° extension to 30° flexion (from −10° to 30°) (47, 48), HIP-ANGLE-C1 and C2 show excessive flexion overall, even when excluding the difference due to HAL usage (Figure 3). The causes of excessive hip flexion include hip flexion contracture and spasticity of the hip flexors (46). However, in individuals with neuromuscular diseases undergoing cybernics treatment, excessive hip flexion is more likely to be caused by muscle weakness, often accompanied by excessive knee flexion and anterior trunk tilt. The pattern in HIP-ANGLE-C1 was rare and cannot be generalized, but it is influenced by low initial gait ability, suggesting a strong possibility of significant progression of muscle weakness. The relationships between the measured items were not examined in this study; therefore, additional research on the possibility of compensatory movements, such as excessive knee flexion and anterior trunk tilt, is needed. The slightly stronger extension during the stance phase in the HIP-ANGLE-C3 (Figure 3) is likely because individuals with higher gait ability can achieve better hip extension. Alternatively, it may be due to the HAL applying more hip torque in the extension direction. Conversely, the larger flexion during the swing phase in the HIP-ANGLE-C5 (Figure 3) may be because the HAL applies more hip torque in the flexion direction.

A noticeable feature of the knee joint angle pattern is the lack of knee extension during the stance phase in the KNEE-ANGLE-C1 and C2 (Figure 4). The gait of people without gait disorders and without HAL has a total of two flexion peaks: one in the stance phase and the other in the swing phase. The knee flexes by approximately 5° at the heel strike (at 0% of the gait cycle), and in the first half of the stance phase, it flexes to approximately 20° at the first peak and then returns to approximately 5° flexion. Subsequently, it reaches approximately 60° of flexion at the second peak during the swing phase, and the flexion angle decreases again toward the end of the gait cycle (43, 47). In the KNEE-ANGLE-C1 and C2, the maximum extension of the average pattern was considerably greater than 5°, which indicates the knee was in hyperflexion. A similar pattern of excessive flexion was observed in the knee joint patterns of individuals with bilateral plantar flexor weakness, as measured by Waterval et al. (49), suggesting that this pattern is likely to occur in those with muscle weakness. The results of the Bayesian regression showed a similar trend, indicating that individuals with lower gait ability are more likely to exhibit excessive knee flexion (Table 3; KNEE-ANGLE-C2). In the gait of individuals using HAL, excessive flexion can be suppressed by strengthening the knee extension assistance. In KNEE-ANGLE-C5, the flexion angle at the second peak during the swing phase is the smallest, but it does not seem to differ much from the maximum flexion angle in normal walking (Figure 4). At C3, the maximum flexion angle was larger (Figure 4), and excessive flexion during the swing phase was thought to compensate for excessive ankle plantar flexion to ensure clearance (46). During HAL intervention, there is also the possibility that the assistive force for knee flexion is too strong. An interesting difference between KNEE-ANGLE-C3 and C5 is that the likelihood of excessive knee flexion in the swing phase increases in individuals with neurogenic diseases, whereas the likelihood of less or normal amounts of knee flexion increases in those with myogenic diseases (Table 3). The mechanisms underlying this phenomenon warrant further investigation.

The differences between clusters in the trunk pitch angle mainly represented the degree of forward or backward tilt of the trunk, particularly in the lumbar region where the HAL control system was located. During normal walking without a HAL, the trunk is tilted forward by approximately 5° (50). The angle varied by cluster; however, in TRUNK-PITCH-C1 through C4, the trunk was tilted forward more than usual (Figure 5). Some researchers have mentioned that forward trunk tilt during walking without HAL is caused by weakness of the hip extensors or quadriceps (51). A similar tilt pattern in the trunk pitch angle may also appear during walking with the HAL, despite the presence of assistive torque. Additionally, using assistive devices with the upper limbs to support the trunk can cause a forward tilt (46). Therefore, if a harness is used to support the body during treatment, the trunk is likely to tilt forward. The results of the Bayesian analysis showed that individuals with myogenic disease and greater decline in gait ability tended to have a more pronounced forward-leaning posture (Table 4; TRUNK-PITCH-C2 and C3). Generally, muscle weakness due to myopathy, except for distal myopathy, is primarily seen in proximal muscles (52). Our results support the hypothesis that individuals with myogenic diseases have difficulty maintaining trunk stability as the disease progresses, resulting in a forward-leaning posture during HAL-assisted walking. On the other hand, individuals in TRUNK-PITCH-C5, C6, and C7, where the trunk does not lean excessively forward and remains upright, tend to have relatively higher gait ability. However, the likelihood of C6 increased when the gait ability was lower within individuals, suggesting that a decline in gait ability may lead to a tendency to lean backward.

Regarding the trunk roll angle, the characteristics are divided into cases where there is a significant left-right amplitude from the equilibrium position (Figure 6; TRUNK-ROLL-C2 -C4) and cases where there is a low amplitude (Figure 6; TRUNK-ROLL-C1 and C5). In low amplitude clusters, such as C1 and C5, the trunk movement was considered restricted. In these clusters, the lumbar region tends to be constantly tilted to either the left or right, which may be caused by scoliosis, a condition that can also occur in patients with neuromuscular diseases (53). Among the clusters with a relatively clear sway, the patterns differed between TRUNK-ROLL-C3 and -C2/-C4. The pattern in C3 was similar to that of the superior iliac spine in the frontal plane of healthy men, as shown in a study by Ceccato et al. (50). However, since the TRUNK-ROLL-C3 pattern was more likely to appear when the 2-min walk distance within an individual was shorter, it must be carefully considered whether the similarity to the pattern in healthy people represents good gait status in gait with HAL. In C2 and C4, the trunk was tilted toward the side opposite the supporting leg during the stance phase, which was related to the neurogenic diseases. This occurs when it is difficult to stabilize the pelvis, such as in cases of muscle weakness in the hip abductor on the same side as the supporting leg (46). Because the HAL does not generate torque to support movement in the frontal plane, it is inferred that in individuals with advanced muscle weakness, pelvic tilt toward the side opposite the supporting leg occurs even while wearing the HAL.

An interesting aspect of the HAL hip and knee joint torque patterns is that, depending on the cluster, the assistive torque acts in different directions (flexion or extension) even with the same gait phase. Regarding hip torque, HIP-TRQ-C2 showed flexion torque even in phases in which the other clusters exhibited extension torque (Figure 7). In terms of knee torque, most clusters exhibited extension torque during the first half of the gait cycle, whereas KNEE-TRQ-C4 exhibited flexion torque (Figure 8). The torque output is determined by the magnitude of the bioelectrical signals from the flexor and extensor muscles, and it is possible to adjust the magnitude and balance of the flexion and extension torque using parameters in the HAL's settings. The difference in amplitude, for example, between HIP-TRQ-C4 and -C5 (Figure 7), was presumably caused by differences in the amplitude of the measured bioelectrical signals or the assist gain in the HAL settings. Since individuals with relatively higher initial gait ability tended to exhibit the HIP-TRQ-C5 pattern, these differences in amplitude may reflect variations in the wearers’ gait abilities. Alternatively, the fact that individuals with neurogenic diseases were more likely to exhibit the HIP-TRQ-C4 pattern than those with myogenic diseases suggests that these differences may be related to the type of disease. Furthermore, the results for KNEE-TRQ-C2 indicate that greater assistive torque is required when gait ability declines. Although we do not currently know the factors that cause differences in the patterns that produce opposite torques to other clusters during certain gait phases, such as HIP-TRQ-C2 and KNEE-TRQ-C4, we believe that it is necessary to understand these differences when assessing the gait during HAL intervention. In addition, the effect of these different torque patterns on the actual joint movements is an interesting research topic.

The clustering results showed asymmetry in the ground reaction forces (Figure 2; GRF-C2 and C4) and the trunk roll angles (Figure 6; TRUNK-ROLL-C1, C2, C4, and C5). The results of the Bayesian regression suggest that myogenic diseases are more likely to cause asymmetry in ground reaction force patterns (Table 1; GRF-C4), while asymmetry in trunk sway appears to be somewhat more pronounced in neurogenic diseases, as seen in one of the four clusters showing asymmetry (Table 5; TRUNK-ROLL-C4). Previous studies on progressive neuromuscular diseases have shown that these diseases sometimes cause asymmetrical muscle weakness and associated bone deformities (3, 54–56). Our clusters revealed that there is often left-right asymmetry in gait when wearing the HAL as well. This study did not specifically examine the asymmetry of the left and right legs, and the clustering results did not explain the asymmetry in the joint angles or joint torques of the HAL. Future work will detail the differences between the left- and right-leg patterns. Analyzing gait patterns while wearing HAL to capture left-right differences would help tailor assistance to the characteristics of each leg and track changes in the degree of asymmetry.

One limitation of our study is that the clustering results depend on the sample data. Considering that a cluster containing data from only one participant was formed in the analysis of the hip joint angle patterns, there is no guarantee that these results are universal. If a pattern that is significantly different from the others emerges, it may not resemble any of the clusters formed in this study. Nevertheless, the fact that we were able to use a larger amount of data, with 137,100 gait cycles from 457 treatment sessions in 48 individuals, compared to previous studies on neuromuscular disease treatment using HAL (2, 13, 14) is a strength of our research. Another potential limitation is that the clusters obtained in our results may not always represent the optimal partitions. We here limited the number of clusters to a range of five to ten to achieve a balance between preventing overgeneralization and ensuring ease of interpretation. Subsequently, we decided on the final number of clusters based on the percentage change in the linkage distance with a change in the number of clusters. Several other methods are present for determining the number of clusters in hierarchical clustering (57) and it is necessary to consider them based on the purpose of the analysis. Furthermore, it is important to interpret our results with the understanding that, although we facilitated the capture of gait pattern features by clustering, we have not discussed whether the patterns in each cluster are desirable in terms of HAL intervention. In the intervention, it is not simply a matter of imitating the desired gait patterns of other clusters.

In this study, we analyzed the data concerning neuromuscular diseases. Our approach is also applicable to other diseases and disorders for which HAL interventions have been applied, such as stroke (58), cerebral palsy (59), and spinal cord injury (60). Because gait disorders vary in nature depending on their cause, it is anticipated that gait patterns will differ based on the specific diseases causing them. Indeed, differences between neurogenic and myogenic diseases were observed in this study. Examining differences in gait patterns due to diseases and disorders will help us better understand gait and implement personalized HAL interventions.

As mentioned in the Introduction, we aimed to establish an advanced system by accumulating and analyzing gait data obtained during HAL-assisted treatment, which contributes to gait evaluation. The importance of our results in sorting the gait patterns during HAL use into several clusters lies in making it easier to capture gait characteristics during cybernics treatment. This has likely become the first step toward effective utilization of the kinematics and kinetics data automatically measured by the HAL.

## 5 Conclusion

We have been developing a pioneering system to monitor the wearer's gait condition associated with cybernics treatment by accumulating and analyzing gait data measured during intervention, rather than relying on conventional gait tests based on distance and speed without HAL, enabling a multi-faceted understanding of the wearer's gait characteristics and condition. As part of this effort, we focused on the gait data automatically measured by HAL during assisted walking and clustered the gait patterns from the cybernics treatment into subgroups. The time-series patterns of the ground reaction forces, lower limb joint angles, trunk angles, and joint torques of the HAL were divided into 5–10 subgroups using a hierarchical clustering method, indicating that the gait patterns during walking with the HAL vary widely with several characteristic types. Moreover, the analysis of Bayesian regression models provided interpretations of how the wearer's disease type and gait ability influenced the differences in the probability of belonging to each subgroup. These differences in gait patterns suggest the diversity and complexity of gait generated through the interaction between the wearer and HAL, which implies the potential for more optimal intervention based on the characteristics captured by such subgrouping, depending on the gait condition. Our research highlights the importance of analyzing and evaluating gait while wearing HAL in cybernics treatment and demonstrates the usefulness of gait data automatically measured by HAL. HAL is expected to function not only as a therapeutic device but also as a gait measurement device for analysis and evaluation. Further work will investigate more detailed and additional factors that produce differences in gait patterns during the intervention and the impact of these differences on treatment efficacy, leading to sophisticated evaluation and feedback in cybernics treatment.

## Data Availability

The data analyzed in this study is subject to the following licenses/restrictions: the data that support the findings of this study are available from CYBERDYNE Inc., but restrictions apply to the availability of these data, which were used under license for the current study, and so are not publicly available. Requests to access these datasets should be directed to nihei_makoto@cyberdyne.
