# Supplementary material for: Analyzing gait data measured by wearable cyborg hybrid assistive limb during assisted walking: gait pattern clustering
Source: Front Med Technol. 2024 Dec 16;6:1448317. doi: 10.3389/fmedt.2024.1448317 (PMC11682894; doi:10.3389/fmedt.2024.1448317)
Supplement: Supplementary file 1 [file Datasheet1.docx]

Supplementary Material A: Segmentation of Gait Cycle

# Algorithm of segmentation

Gait is a cyclic motion, and gait analysis typically deals with data segmented into cycles that start and end with the heel strike. In cybernics treatment for neuromuscular diseases, the walkers undergoing treatment may struggle to lift their feet sufficiently off the ground, causing the boundary between the swing and stance phases to be unclear. Additionally, their gait may involve landing with the toe side rather than the heel. To accommodate these types of gaits, we adopted a method for detecting cycles that uses the ratio of the load on the left and right feet.

From the measurements of the plantar load sensors in HAL shoes, the ratio of the load $W_{\mathrm{LR}}$, proposed by Hayashi *et al.*[27], was calculated as follows:

$$\begin{aligned} W_{\mathrm{LR}}=\frac{\sum_{i} f_{R_{i}}-\sum_{i} f_{L_{i}}}{\sum_{i} f_{R_{i}}+\sum_{i} f_{L_{i}}}, \end{aligned}$$

where $f$ are the plantar loads from each sensor, the subscripts L and R represent the left and right legs, and $i$ describe the channels of sensors. During gait, the value of $W_{\mathrm{LR}}$ varies cyclically in response to the body movement in the frontal plane. Within each gait cycle, $W_{\mathrm{LR}}$ takes its maximum value when the right leg is supporting the body and its minimum value when the left leg is supporting the body. The difference between the maximum and minimum values of $W_{\mathrm{LR}}$ was defined as the range of load transfer. The start of the right leg’s gait cycle was identified as the point where $W_{\mathrm{LR}}$ decreased by an amount equal to 5% of this range from its maximum value, moving backward in time from the point at which $W_{\mathrm{LR}}$ reached its peak (Supplementary Figure 1). Similarly, the start of the left leg’s gait cycle was identified as the point where $W_{\mathrm{LR}}$ increased by 5% of the range of load transfer, moving backward in time from the point at which $W_{\mathrm{LR}}$ reached its minimum value.

# Definition of reference leg in a gait cycle

Gait is a movement in which the right and left legs alternately perform periodic motions with approximately half a cycle difference. Thus, a gait cycle can begin either with right heel contact or left heel contact. In this study, we analyzed time-series data from both legs. To analyze both legs within a common framework that eliminates the half-cycle difference in gait phase, we defined the leg of interest during each gait cycle as the reference leg. Specifically, in a gait cycle that begins with the right leg contact, the right leg was treated as the reference, while the left leg was considered the opposite leg. Conversely, in a gait cycle that begins with the left leg contact, the left leg was treated as the reference leg, and the right leg as the opposite leg (Supplementary Figure 2).


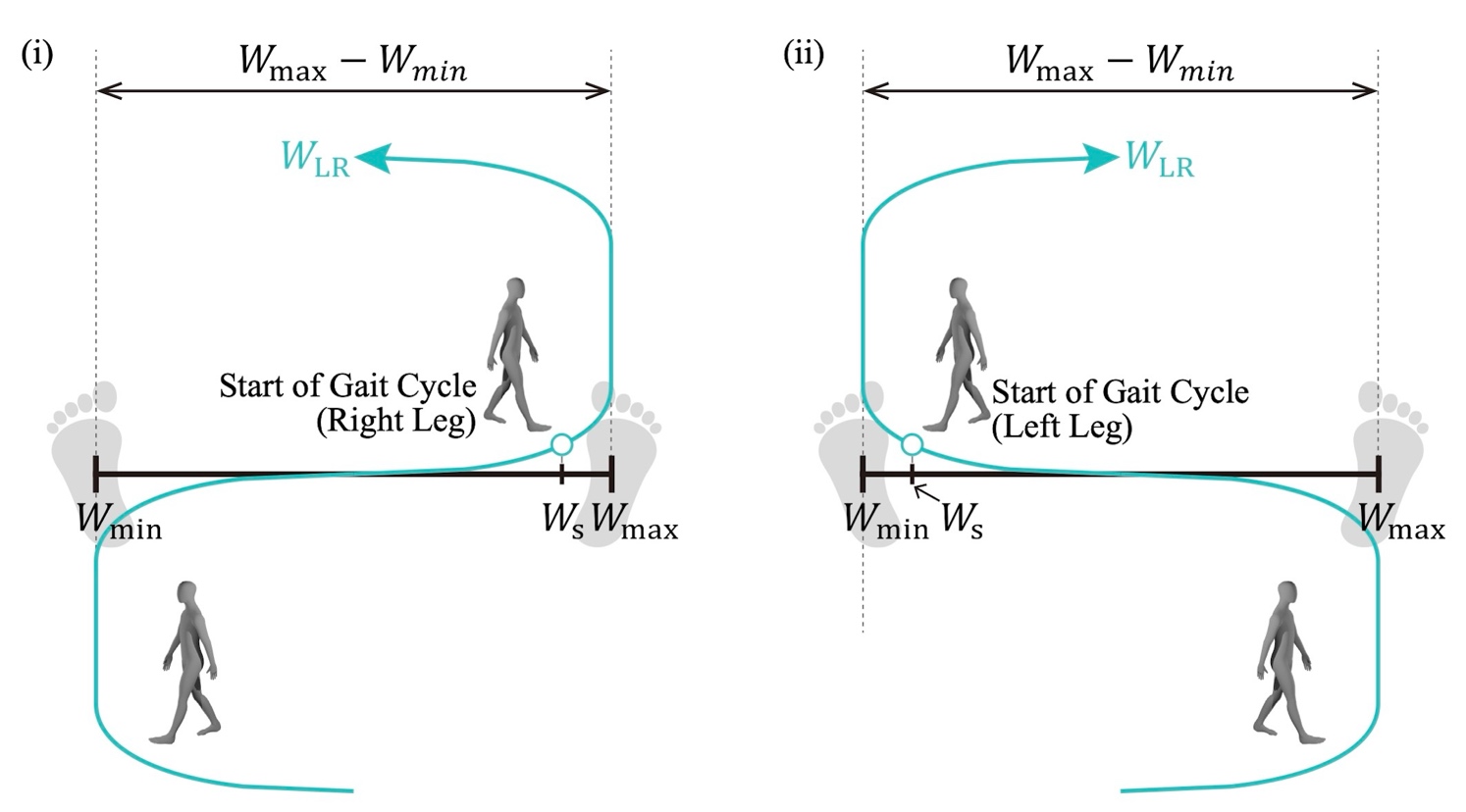


**Supplementary Figure 1.** Method of gait cycle segmentation using left-right load ratio. The value of $W_{\mathrm{LR}}$ varies cyclically in the range of –1 to 1 in response to the gait motion (cyan solid line). (i) The start of the right leg’s gait cycle was defined as the point $W_{s}=W_{\max}-0.05(W_{\max}-W_{\min})$, with $W_{\max}$ as the maximum and $W_{\min}$ as the minimum of $W_{\mathrm{LR}}$ within the cycle. (ii) Similarly, the start of the left leg’s gait cycle was defined as the point $W_{s}=W_{\min}+0.05(W_{\max}-W_{\min})$.


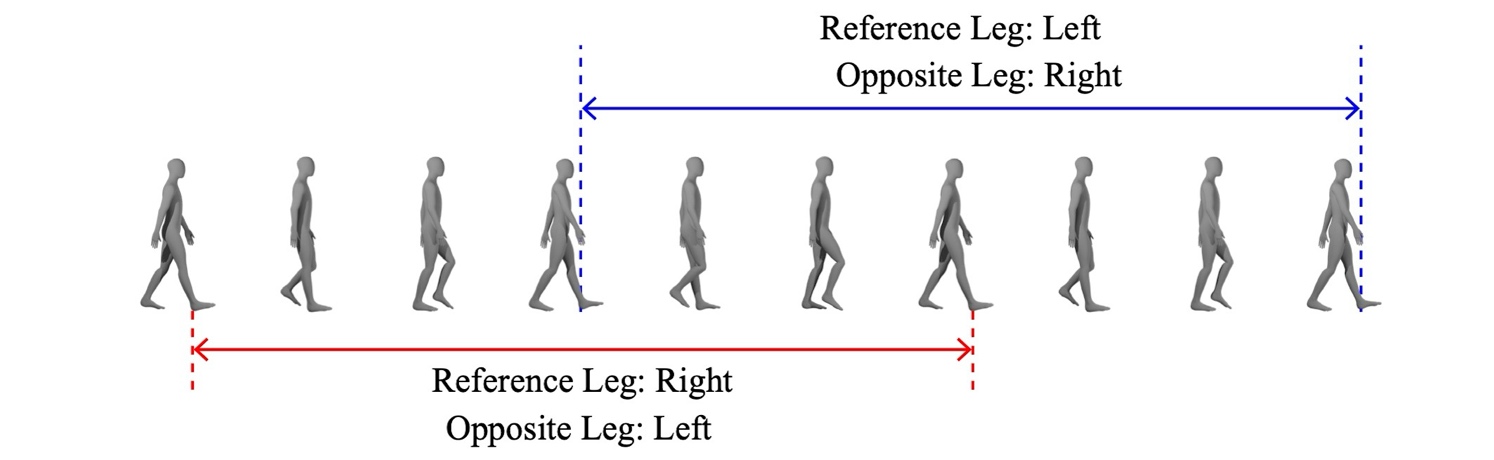


**Supplementary Figure 2.** Explanation of the reference leg. The gait cycle indicated by the red line begins with the initial contact of the right leg, so the right leg is treated as the reference leg. The gait cycle indicated by the blue line begins with the initial contact of the left leg, so the left leg is treated as the reference leg.
